# Present and Future: Crosstalks Between Polycystic Ovary Syndrome and Gut Metabolites Relating to Gut Microbiota

**DOI:** 10.3389/fendo.2022.933110

**Published:** 2022-07-19

**Authors:** Mingmin Zhang, Runan Hu, Yanjing Huang, Fanru Zhou, Fan Li, Zhuo Liu, Yuli Geng, Haoxu Dong, Wenwen Ma, Kunkun Song, Yufan Song

**Affiliations:** ^1^ Department of Integrated Traditional Chinese and Western Medicine, Tongji Hospital, Tongji Medical College, Huazhong University of Science and Technology, Wuhan, China; ^2^ Institute of Integrated Traditional Chinese and Western Medicine, Tongji Hospital, Tongji Medical College, Huazhong University of Science and Technology, Wuhan, China

**Keywords:** polycystic ovary syndrome, gut metabolites, crosstalk, bile acids, short chain fatty acids, amino acids

## Abstract

Polycystic ovary syndrome (PCOS) is a common disease, affecting 8%–13% of the females of reproductive age, thereby compromising their fertility and long-term health. However, the pathogenesis of PCOS is still unclear. It is not only a reproductive endocrine disease, dominated by hyperandrogenemia, but also is accompanied by different degrees of metabolic abnormalities and insulin resistance. With a deeper understanding of its pathogenesis, more small metabolic molecules, such as bile acids, amino acids, and short-chain fatty acids, have been reported to be involved in the pathological process of PCOS. Recently, the critical role of gut microbiota in metabolism has been focused on. The gut microbiota-related metabolic pathways can significantly affect inflammation levels, insulin signaling, glucose metabolism, lipid metabolism, and hormonal secretions. Although the abnormalities in gut microbiota and metabolites might not be the initial factors of PCOS, they may have a significant role in the pathological process of PCOS. The dysbiosis of gut microbiota and disturbance of gut metabolites can affect the progression of PCOS. Meanwhile, PCOS itself can adversely affect the function of gut, thereby contributing to the aggravation of the disease. Inhibiting this vicious cycle might alleviate the symptoms of PCOS. However, the role of gut microbiota in PCOS has not been fully explored yet. This review aims to summarize the potential effects and modulative mechanisms of the gut metabolites on PCOS and suggests its potential intervention targets, thus providing more possible treatment options for PCOS in the future.

## 1 Introduction

Polycystic ovary syndrome (PCOS), characterized by oligo-ovulation or anovulation, hyperandrogenemia, and polycystic ovarian morphology, is a common disorder of the reproductive endocrine system, affecting 8%–13% of the women of reproductive age as well as impairing their fertility and long-term health (1, 2). Women with PCOS have a higher risk of infertility and pregnancy complications, accompanied by subsequent complications, such as obesity, type 2 diabetes, non-alcoholic fatty liver disease (NAFLD) (3), cardiovascular disease (4), endometrial cancer, and osteoporosis (5). All these complications have a far-reaching impact on the physical and mental health of women (6).

Until now, the specific etiology and pathophysiology of PCOS remain unclear. PCOS might be a polygenic heritable condition, which is affected by a variety of acquired variables (7). Hyperandrogenemia is generally regarded as the core part of PCOS, causing reproductive disorders, insulin resistance (IR), and metabolic imbalances, such as glucose and lipid metabolic imbalance (8). In particular, the IR and compensatory hyperinsulinemia might cause abnormality in the sex hormone levels, chronic inflammation, and metabolic disorders, thereby contributing to follicular dysplasia (9). These pathological factors create a vicious cycle, which increases the obstacles to PCOS treatment.

With a deeper understanding of gut biology, the potential role of the gut in PCOS has become the center of attention. Gut microbiota, also known as the “second genome” of the host, can affect the metabolism and immune response of the host by interacting with the external environment (8). Alpha-diversity (α-diversity) is regarded as an indicator of ecosystem health, representing the number of species present in the given community, whereas beta-diversity (β-diversity) denotes the similarity of a community or individual sample with another community or individual sample, respectively (10). As compared to the normal group, the dysbiosis of gut microbiota in the PCOS women showed lower α- and β-diversities, decreased relative abundance of *Bifidobcterium*, and increased relative abundances of *Bacteroides*, *Parabacteroides*, and *Clostridium* (11–13). Furthermore, the dehydroepiandrosterone (DHEA)–induced PCOS rats showed the dysbiosis of gut microbiota, and transferring this microbiota to healthy rats could induce the PCOS-like metabolic and endocrinal dysfunctions, indicating that the gut might be a novel therapeutic target for the treatment of PCOS (14).

Recently, studies on gut metabolites have emphasized the importance of the gut in maintaining general homeostasis. The gut metabolites, such as bile acids (BAs), amino acids, and short-chain fatty acids (SCFAs), are greatly involved in modulating the integrity of the gut barrier, thereby maintaining the internal environment and homeostasis. A disturbance in gut metabolites might increase the gut permeability, leading to the leakage of lipopolysaccharides (LPSs) and endotoxemia, which might disturb the endocrine system, immune system, insulin signaling, glucose metabolism, lipid metabolism (8), and gut microbiota (15). Furthermore, the SCFAs, BAs, and branched-chain amino acids (BCAAs) can directly regulate the secretion and sensitivity of pancreatic insulin in the target organs through endocrine signaling. While circulating through the portal venous system, these metabolites reach the liver to regulate lipid metabolism and oxidation. Moreover, these metabolites also take part in neuronal homeostasis by modulating the integrity of the blood–brain barrier (16). The gut–brain peptides, which can be affected by gut metabolites, might communicate with the brain, thereby influencing appetite and energy maintenance as well as increasing the secretion of luteinizing hormone (LH) (17).

Interestingly, the activity and contents of gut metabolites can be regulated by the gut microbiota (18, 19). The correlations between gut metabolites and gut microbiota have been demonstrated in numerous metabolic diseases, such as obesity, type 2 diabetes, NAFLD, and cardiovascular diseases (20, 21). Qiao and colleagues also demonstrated that an increase in the relative abundance of *Bacteroides* in patients with PCOS was related to the disturbance in gut metabolites, which might have a potential pathological role in PCOS (13, 22).

These studies indicate that, in PCOS, the gut microbiota and related metabolites might be affected. They both are closely linked to the insulin signaling pathway, steroid hormone levels, glucose metabolism, lipid metabolism, and immunological homeostasis, all of which are greatly involved in the pathogenesis of PCOS (16, 17). However, understanding the mechanism of interactions between gut metabolites and PCOS is still unclear. This review aims to summarize the existing studies and demonstrates the interaction between PCOS and gut microbiota-related metabolites, which might help in developing novel treatments for PCOS.

## 2 BAs

### 2.1 Biosynthesis and Metabolism of BAs

BAs are the key metabolites, which include primary BAs and secondary BAs. In humans, cholic acid (CA) and chenodeoxycholic acid (CDCA) are the most common primary BAs. Under normal physiological conditions, these primary BAs are synthesized from cholesterol in the pericentral hepatocytes through “classical” (neutral) and “alternative” (acidic) pathways (23, 24). The classical pathway favors the biosynthesis of CA and CDCA (25), whereas the alternative pathway only favors the biosynthesis of CDCA. After the primary BAs are modified and transported by various enzymes and transporters, they are conjugated with taurine or glycine and secreted into the bile, which are then released into the small intestine and aid in lipid digestion (26). In the ileocecum, the gut microbiota and bile salt hydrolase (BSH) convert the conjugated BAs to free BAs. Following the modifications, such as the removal, oxidation, or epimerization of the nuclear hydroxyl by the host or gut microbiota, the free primary BAs are converted into secondary BAs (25, 27). The secondary BAs play a critical role in regulating glucose metabolism, insulin signaling, lipid metabolism, and inflammation. In the distal ileum, most of the secreted molecules (95%) are reabsorbed through apical sodium-dependent BA transporter (ASBT) and are ultimately transported into the liver through the portal vein system. This phenomenon is known as enterohepatic circulation. Meanwhile, the remaining secreted molecules are excreted in feces (15, 25). In the ileum, BAs facilitate the secretion of fibroblast growth factor 19 (FGF19) in humans or FGF15 in mice by activating the farnesoid X receptor (FXR). FGF19 further represses the BA synthesis as negative feedback when circulated to the liver (26).

The above process relies heavily on gut microbiota. The gut microbiota can affect the production of BAs by regulating the liver enzymes, such as 7a hydroxylase and sterol-27-hydroxylase, especially CDCA in humans (28). BSH has been widely detected in rodent and human gut microbiota, such as *Clostridium* spp (29).. The diversity of secondary BAs is greatly affected by the species differences in gut microbiota (23). The mouse models in the absence of gut microbiota demonstrate that almost all the BAs were primary BAs, indicating the importance of gut microbiota in the production of free BAs (15, 30). The gut microbiota could affect the ileum mucosa and ASBT to regulate the reabsorption of BA in rodents (28). Moreover, the gut microbiota partially inhibits the BA biosynthesis through the FXR-dependent mechanism (31–33). The BAs shape the structure of gut microbiota and exert antibacterial effects by selectively promoting the growth of BA-synthesizing bacteria, thereby showing a bidirectional communication between the gut microbiota and BAs (15, 33).

### 2.2 Role of BAs in Metabolism, Endocrine, and Inflammation

One of the most important functions of BAs is their participation in lipid emulsification and solubilization. They convert fat into fat droplets, which can be digested by trypsin and absorbed by gut mucosa, thereby assisting the absorption of dietary fat, which is critically important for lipid metabolism (34). Certain essential vitamins, such as vitamins A and D, are non-polar lipids, which can only be absorbed if bound to micelles in the presence of BAs (25, 35). Whenever the concentration of cholate is lower, cholesterol absorption is inhibited (25).

The BAs modulate metabolic homeostasis by stimulating the receptors, such as G protein receptor 5 (TGR5) and FXR. TGR5 is widely distributed in a variety of animal tissues, such as fat, central nervous system, liver, and gut and participates in regulating insulin signaling, glucose metabolism, and energy expenditure in brown adipose tissue and muscle (36). The intestinal hormone glucagon-like peptide 1 (GLP-1) and peptide YY (PYY) are simulated by TGR5 (37). In the murine brain, BAs could activate TGR5, causing the central anorexigenic actions to control the appetite (38). FXR is another BAs receptor, which is found in white adipose tissue, liver, gut, immune cells, and other tissues (39). The BAs, in combination with FXR, can induce FGF15 and/or FGF19, which might regulate glucose tolerance and normal glycemia by reducing hepatic gluconeogenesis. A reduction in the number of activated FXR might reduce the secretion of FGF15 and/or FGF19. This might result in the increase of hepatic gluconeogenesis, deposition of hepatic lipid, and disruption of glucose homeostasis in adipocytes and the decrease of insulin production in pancreatic cells (26, 32). Moreover, in the cardiac and visceral fat cells, tauroursodeoxycholic acid (TUDCA) could reduce endoplasmic reticulum stress, thereby preventing obesity and inflammation (40). Therefore, reduction in the TUDCA might result in the diminished suppression of abdominal and visceral fat inflammation, aggravating the IR and metabolic disorders (8, 40).

However, the current studies on the link between BAs and metabolic syndrome include a few individuals in the BAs pool. The levels of fasting circulating total BAs were higher among the populations with mild IR and obesity (25). However, the changes in the levels of fasting circulating BAs in disease states as well as the role of each BA in metabolic diseases are needed to be investigated.

## 3 SCFAs

### 3.1 Biosynthesis and Metabolism of SCFAs

Recently, the crosstalk between SCFAs and gut has been focused on. The SCFAs originated from the microbiota-accessible carbohydrates (MACs) in the colon (41), which are fermented from the dietary fibers and resistant starch ferment. They mainly consist of acetic, propionic, butyric, valeric, and caproic acids, which are biosynthesized in various pathways, such as the Wood–Ljungdahl pathway, aided by the different classes of gut microbes (42). The exact contents and relative proportion of each type of SCFA might differ based on the diet, composition of the microbiota, and gut transit time (43, 44). When the BCAAs, including valine, isoleucine, and leucine, escape digestion in the upper gut, they might be fermented into branched-chain fatty acids (43, 44). Furthermore, the SCFAs are taken up by colonocytes *via* passive diffusion or active transport (42). A part of the unmetabolized SCFAs are transported into the liver through the portal system and serve as substrates for the energy metabolism and anabolic processes, thereby playing a prominent role in the inhibition of glycolysis, stimulation of lipogenesis and gluconeogenesis, and regulation of mitochondrial energy production (45).

### 3.2 Role of SCFAs in Metabolism, Endocrine, and Inflammation

SCFAs are important for balancing metabolism and energy. They are taken up by colon cells after binding to G protein–coupled receptors (GPCRs), which are also known as free fatty acid receptors (FFARs) and are present on the enteroendocrine cells of the gastrointestinal mucosa (46), thereby stimulating the secretion of intestinal hormones, such as GLP-1, PYY, gamma-aminobutyric acid (GABA), and serotonin (5-HT) (46). The intestinal hormones aid in reducing the production of hepatic glucose, enhancing the absorption of peripheral glucose, and suppressing the appetite (47). Moreover, the SCFAs can also stimulate leptin secretion in adipocytes and insulin secretion in the pancreatic cells (48). The circulating SCFAs can activate the burning of brown adipose tissue, thereby increasing energy consumption and preventing weight gain (43, 49). In addition, the SCFAs also improve insulin sensitivity in the muscle and liver tissues (47).

In contrast to hepatic gluconeogenesis, intestinal gluconeogenesis (IGN) is beneficial for controlling the glucose level by reducing food intake and hepatic glucose output (47, 49). In a study based on mouse models, butyrate could directly promote the IGN expression in enterocytes in a Cyclic Adenosine Monophosphate (cAMP)-dependent manner, whereas propionate could increase the IGN expression by binding to FFAR3 in the portal nerve, thereby initiating the portal-hypothalamic crosstalk, improving the insulin sensitivity and glucose tolerance, and lowering the fat mass (47).

Recently, studies have demonstrated that SCFAs could affect the host’s immune system. SCFAs could affect the hematopoietic progenitors in the murine bone marrow, implying that they were important for the development of innate and adaptive immune systems (50). Moreover, they exerted a systematic anti-inflammatory effect in mice by affecting the peripheral DCs and T cells (51). In particular, the SCFAs increased the number of T-regulatory (Treg) cells, induced the differentiation of Treg cells, and regulated the production of interleukin, thereby minimizing the oxidative stress and protecting pancreatic cells (52–54). In a murine model of gout, SCFAs could bind to the GPCR43 in the central nervous system and act on microglia to regulate host immunity (55). Furthermore, they strengthened the integrity of the blood–brain barrier and regulated the levels of neuronal factors and neurogenesis to relieve the neural and central inflammation (52).

Moreover, SCFAs can also increase the expression of intestinal epithelial tight junction protein and decrease the death of intestinal epithelial cells (IEC), thereby promoting gut mucosal immunity and barrier integrity (56, 57). Once the intestinal mucosal barrier is disrupted, LPS enters the blood circulation, resulting in a persistent inflammation, which is correlated with IR (51).

The SCFAs, when reaching the brain, can alter the integrity of the blood–brain barrier by increasing the expression of tight junction proteins in the blood–brain barrier and regulating the state of neural and central inflammation (41, 52). SCFAs also affect the function of glial cells and neurogenesis in order to maintain neuronal homeostasis (41).

## 4 Amino acids

### 4.1 Anabolism and Catabolism of Amino Acids

Amino acids, consisting of essential and non-essential amino acids, are life-supporting molecules, which provide raw materials for protein synthesis. The food amino acids are primarily absorbed in the small intestine *via* the concentrative amino acid transporters. A small number of amino acids are also absorbed by the large intestine, whereas the remaining are excreted in the feces (58, 59). Then, the amino acids are released primarily through passive efflux across the basolateral membrane, which is mediated by a group of transporters (59, 60). When released into the bloodstream, they are transported into the cells *via* the corresponding secondary active transporters, which are also called functional transporters (61). Simultaneously, an increase in the cytoplasmic amino acid pool activates the amino acid metabolism, forcing the excess amino acids to be catabolized *via* oxidation, hydroxylation, and other processes (62). The majority of amino acids are metabolized and restored in the liver (58). However, they may also be stored in extrahepatic tissues, such as muscle, brown fat, kidneys, liver, and heart tissues (62); this storage in extrahepatic tissues is regulated by the insulin-mediated signaling in the hypothalamus (63).

### 4.2 Role of Amino Acids in Metabolism, Endocrine, and Inflammation

In addition to synthesizing proteins, amino acids are also involved in glycolysis and mitochondrial metabolism through the tricarboxylic acid (TCA) cycle and oxidative phosphorylation and modulate the cellular activities, such as lipid and glucose metabolism (64). By acting on the IR substrates (IRSs), the amino acids can affect insulin signaling (65). Furthermore, recent studies have demonstrated that amino acids are the potential precursors of the brain neurotransmitter, impacting habits (66). Furthermore, amino acids participate in ATP generation, nucleotide synthesis, and redox balance, which support the growth, proliferation, and effector function of immune cells (64, 67).

## 5 Crosstalk between PCOS and gut metabolites

### 5.1 Effect of PCOS on Gut Metabolites

To date, significant differences have been reported between the gut microbiota and metabolites in patients with PCOS as compared to the control group. PCOS, as a multi-system endocrine disease, has a negative impact on the function and composition of gut microbiota and metabolites.

#### 5.1.1 Impact of Sex Hormones on Gut Microbiota and Related Metabolites

According to studies, sex hormones have a substantial impact on the composition of the gut microbiota. They affect the composition of gut microbiota in a sex-specific manner after puberty (10, 68). As a result, the gut microbiota in females has higher α-diversity but significantly lower abundances of *Bacteroides* species, including *Prevotella* and *Bacteroides thetaiotomicron*, as compared to that of males. The studies of rodents and other species have also shown similar results, but the outcomes vary across the studies (68, 69). Meanwhile, the dysbiosis of gut microbiota in the PCOS women was characterized by the lower α-diversity, decreased relative abundance of *Bifidobacterium*, increased relative abundance of *Bacteroides*, and changes in the β-diversity as compared to the control group (11–13). This showed that the gut microbiota of the PCOS women altered when compared to that of the men. Although different conclusions have been presented, the accumulating data confess that hyperandrogenism might affect the gut microbiota of patients with PCOS by affecting the gut function and regulating the activity of β-glucuronidase and its substrate levels, such as bilirubin, neurotransmitters, and hormones, which are present in the liver (10, 11, 70). Because the gut metabolites are closely related to the gut microbiota, the changes in gut microbiota in response to hormones might also change the gut metabolites. For example, Sherman et al. revealed that the prenatal androgens were linked to the changes in the abundance of gut microbiota involved in the production of SCFAs in the rat (71). In a nutshell, the disruption of sex hormones in PCOS affects the composition of gut metabolites and microbiota.

#### 5.1.2 Impact of Obese on Gut Microbiota and Related Metabolites

The dysbiosis of gut microbiota is correlated with the phenotype of PCOS. There are differences in the gut microbiota of non-obese and obese individuals with PCOS (17). The abundance of clostridium cluster XVII increased in the non-obese patients with PCOS, whereas that of *Clostridium sensustricto* and *Roseburia* decreased (72). Liu et al. reported that the relative abundances of gut microbiota, including *Bacteroides*, *Escherichia/Shigella*, and *Streptococcus* increased, whereas those of *Akkermansia* and *Ruminococcaceae* decreased in the patients with PCOS, which were correlated with body mass index (BMI) (17). It has also been reported that obese women with PCOS tend to have lower α-diversity and biodiversity of the gut microbiota as compared to the women with normal BMI (69). Furthermore, according to Li and colleagues, obesity was associated with the altered BA metabolism caused by the dysbiosis of gut microbiota (21). Because adiposity is a source of sex steroids, it might affect the composition of gut microbiota and gut metabolites by affecting the production of sex hormones (73). In addition, obesity might contribute to the development of a chronic inflammatory state, which might alter the gut permeability and microbiota, thereby affecting the function and composition of gut metabolites (21, 74).

#### 5.1.3 Impact of IR on Gut Microbiota and Related Metabolites

Among the women with IR, the relative abundance of *Bacteroidaceae* increased, whereas that of *Prevotellaceae* decreased as compared to the PCOS women without IR (71). The IR-induced gut dysbiosis could result in the accumulation of BCAAs (13, 36, 75, 76). On the other hand, the TCA cycle was significantly inhibited by IR, resulting in decreased BCAA clearance (77). Furthermore, IR and compensatory hyperinsulinism contributed to hyperandrogenism, thereby disrupting the gut dysbiosis and metabolites (8, 10).

#### 5.1.4 Impact of Habits on Gut Microbiota and Related Metabolites

Numerous patients with PCOS have bad habits, such as an adoration of sweets, a love of fat, an absence of dietary fiber, and little exercise, which affects gut health (18, 78). A high-fat diet (HFD) was linked to an increase in the pro-inflammatory microbiota, such as *Clostridiales*, *Bacteroides*, and *Enterobacteriales*, and a decrease in the anti-inflammatory microbiota, such as *Lactobacillus*, in the rat (75). The high levels of glucose, fructose, and sucrose could increase the relative abundance of *Bifidobacteria* while suppressing that of *Bacteroides* (75). The lack of dietary fiber might result in a decrease in the production of SCFAs. All these could affect the biosynthesis of gut metabolites.

Exercise can enhance gut health by increasing the diversity of gut microbiota and balancing the beneficial and pathogenic bacterial communities (79). Specifically, exercise increases the ratio of butyrate-producing bacteria, such as *Roseburia hominis*, thereby increasing the concentration of butyrate (80, 81). Moreover, exercise can reduce the contact time between feces and the gastrointestinal mucus layer by enhancing gastrointestinal motility to benefit gut health (79, 80). It also boosts the production of key antioxidant enzymes and anti-inflammatory cytokines in the intestinal lymphocytes, thereby reducing intestinal inflammation (76, 77). A reduction in exercise might disrupt the gut metabolites (43, 44, 79).

In a nutshell, the dysbiosis of gut microbiota is caused by unhealthy habits, hyperandrogenism, obesity, hyperinsulinism, and disturbances in the glucose and lipid metabolism in PCOS, leading to increased gut permeability, exaggerated dysbiosis, and altered gut metabolites (82, 83).

### 5.2 Effect of Gut Metabolites on PCOS

#### 5.2.1 Effect of BAs on PCOS

The BA metabolism is a key metabolic pathway affected by the changes in gut microbiota in patients with PCOS. Zhang and colleagues demonstrated that an increase in the circulating conjugated primary BAs was positively correlated with hyperandrogenism in women with PCOS (84). In both the stool and serum, the levels of secondary BAs, such as glycodeoxycholic acid (GDCA) and TUDCA, were lower in the PCOS group as compared to those in the control group and were correlated with the disturbance of gut microbiota (13).

The findings from PCOS rats revealed that UDCA administration could improve ovarian morphology and decrease the total testosterone and insulin levels. However, the lipid parameters, E1, E2, glucose, and homeostatic model assessment for IR were comparable between the groups (85).

Moreover, BAs could also regulate the performance of gut immune cells. Both the protein and mRNA levels of Interleukin-22 (IL-22) in the cultured group 3 innate lymphoid cells (ILCs) were greatly stimulated in the presence of TUDCA or GDCA, which was also confirmed in mouse models, showing that TUDCA or GDCA therapy could enhance the mRNA levels of gut IL-22 and alleviate the disease symptoms (13). These beneficial effects of BAs on IR and ovarian function in PCOS mice were reversed by knocking out the *IL-22 receptor* gene (13). The IL-22 levels in serum and follicle fluid of patients with PCOS decreased, whereas the IL-22 administration could improve IR, ovarian dysfunction, dysbiosis of gut microbiota, and prenatal Müllerian hormone in the DHEA-induced PCOS mice (86). Therefore, it could be proposed that the regulatory effects of BAs on PCOS were at least partially mediated by IL-22 (13).

IL-22 has diverse benefits, such as improving insulin sensitivity and regulating the lipid metabolism in the liver and adipose tissues. IL-22 can promote the proliferation of IEC and the production of antimicrobial peptides and mucins in IEC (13, 51). Therefore, a reduction in the IL-22 level might further disrupt the integrity of the gut barrier and microbiome hemostasis, thereby aggravating the endotoxemia, chronic inflammation state, and particularly the IR (13, 87, 88). Recently, Qi et al. reported that IL-22 could reverse the disturbed menstrual cycle in PCOS mice by relieving the inflammatory state in ovarian granulosa cells, further indicating the possible role of IL-22 in the PCOS intervention (86).

#### 5.2.2 Effect of SCFAs on PCOS

SCFAs are essential elements in maintaining the homeostasis of gut microbiota and regulating the intestinal mucosal barrier as an important energy source for the gut microbiota and IEC (51, 89). For instance, butyrate regulates the utilization of intestinal oxygen, thereby regulating the proportion of aerobic and anaerobic gut microbiota. Therefore, a reduction in the SCFA levels might cause gut dysbiosis in PCOS (90) and disrupt the intestinal mucosal barrier to exacerbate the chronic inflammatory state (51).

In addition to affecting the gut health and microbiota, the SCFAs also exert various physiological effects through IL-22. SCFAs could promote the IL-22 production in CD4^+^ T cells and ILCs by binding to the histone deacetylase inhibitors and GPCRs (89). The IL-22 could maintain metabolic homeostasis, which was disturbed by SCFAs reduction (89), thereby producing a synergistic effect with BA–IL-22. In addition, studies reported that the secretion of gastrointestinal hormones in patients with PCOS was disturbed, such as a decrease in the GLP-1 level (91). SCFAs could stimulate the secretion of gastrointestinal hormones, such as GLP-1, PYY, GABA, and 5-HT, thereby reversing their decreased levels in the patients with PCOS, maintaining insulin homeostasis, and suppressing the appetite (46).

In addition to their effects on the gut, SCFAs also exert peripheral effects. SCFAs could promote the *IGN* gene expression in enterocytes through the portal-hypothalamic circuit, thereby maintaining food intake and hepatic glucose output in mice (49). The decrease in the SCFA levels can boost insulin secretion from the pancreatic cells *via* GPCRs, improve insulin sensitivity, increase the energy expenditure in brown adipose tissue, and upregulate the antilipolytic activity of glucose transporter type 4, thereby further aggravating the PCOS (41, 45, 47).

Moreover, Lin et al. discovered that the absorption of SCFAs decreased in the PCOS rats. They demonstrated that the fecal SCFA concentrations increased and were positively correlated with the tumor necrosis factor and IL-6 levels (92). Enhancing the SCFA absorption could improve the integrity of the intestinal mucosal barrier and inhibit intestinal and parenteral inflammation (92).

Therefore, SCFAs are critically important for maintaining glucose and insulin homeostasis and ameliorating chronic inflammation throughout the body. The supplementation of SCFAs or enhancing their beneficial effects, such as activating the relevant receptors, might be helpful in the PCOS treatment.

#### 5.2.3 Effect of Amino Acids on PCOS

Recently, the correlations between BCAAs and metabolic dysbiosis have been focused on. The human body cannot synthesize BCAAs, which are essential amino acids; therefore, they must be absorbed from the digestion of food (65). By phosphorylating the IRS-1 and IRS-2 at serine or damaging the mitochondrial function in the β-pancreatic cells, excessive BCAAs could aggravate IR in rodents with PCOS (65, 88, 93). Furthermore, BCAAs might induce the expression of proinflammatory genes to deteriorate chronic inflammation, thereby developing IR (65).

As compared to the SCFAs and BAs, the current studies on intestinal amino acids are limited. Moreover, the conclusions are not completely consistent due to the inter- and intra-species differences and experimental conditions. The correlations between amino acids and PCOS are still unclear. However, it could be concluded that downregulating the excessive BCAAs or blocking their associated binding sites might further ameliorate IR in PCOS. Whether and how BCAAs can be applied for the prediction and treatment of PCOS are worth exploring.

## 6 Prospects and implications

Currently, the primary goal of PCOS treatment is to alleviate its symptoms, such as hyperandrogenism, IR, oligo- or anovulation, and infertility. For example, letrozole aids in developing the dominant follicles, whereas metformin is typically for the treatment of metabolic symptoms and IR, possibly restoring their ovulation (94). These treatment strategies can only provide temporary relief from the symptoms or achieve a short-term goal. Therefore, the fundamental and permanent treatment of the pathological processes in PCOS is worth exploring.

**Figure 1:**
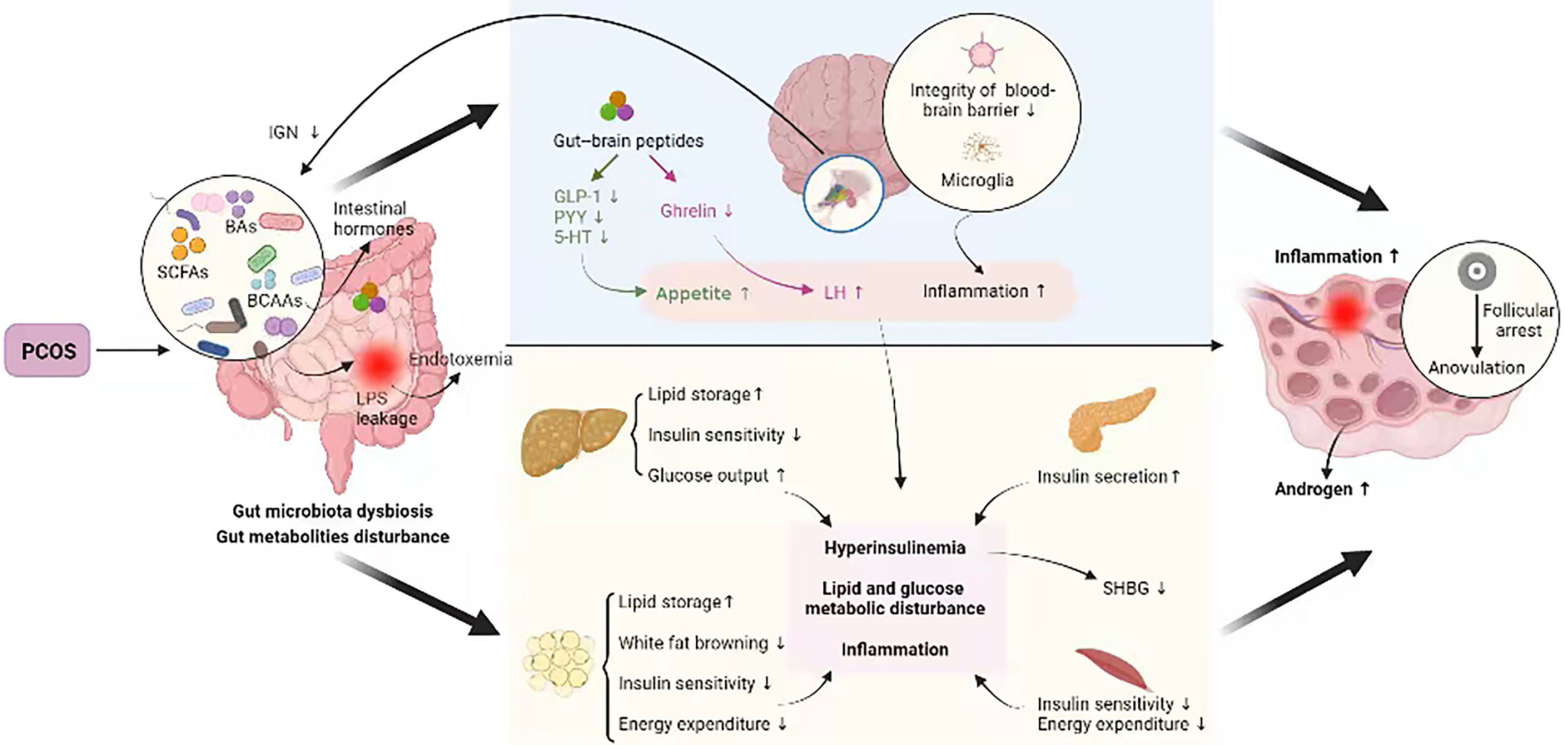
Crosstalk between PCOS and gut metabolites. PCOS, polycystic ovary syndrome; BAs, bile acids; SCFAs, short-chain fatty acids; BCAAs, branched-chain amino acids; IGN, intestinal gluconeogenesis; LPS, lipopolysaccharide; GLP-1, glucagon-like peptide 1; PYY, peptide YY; 5-HT, 5-hydroxytryptamine; LH, luteinizing hormone; SHBG, sex hormone–binding globulin. PCOS disturbs intestinal microbial homeostasis and metabolites, which may be linked to the insulin signaling pathway, steroid hormone levels, glucose metabolism, lipid metabolism, and immunological homeostasis etc., all of which are involved in PCOS pathogenesis, thus forming a vicious cycle.

As stated above, the PCOS-related hyperandrogenism, IR, obesity, metabolic disturbance, unhealthy diet, and other factors could disrupt the gut microbiota and metabolites, which, in turn, deteriorated the pathological process of PCOS, forming a vicious cycle (95) (**Figure 1**).

Interestingly, the changes in gut metabolites could predict PCOS (23, 96) and might even be associated with the different clinical phenotypes (17). The gut metabolites could be more precise predictors than the gut microbiota due to the susceptibility of gut microbiota to a variety of factors, such as environmental contamination and abrupt changes in diet.

The therapies, targeting the gut homeostasis to break the vicious circle between hyperandrogenism and metabolic abnormalities, can be the tipping point for the treatment of PCOS. For example, adopting a healthier lifestyle; supplementing the specific BAs (TDUCA and GDCA), SCFAs, and IL-22; regulating the metabolism of amino acids; and blocking the BCAA targets could be beneficial for the treatment of PCOS (89, 95). It has been reported that the IL-22 levels in patients with PCOS were significantly lower than those in the normal group (22). Therefore, the IL-22 supplementation could be an effective treatment option for PCOS. Studies on the PCOS mice confirmed that the intraperitoneal injection of IL-22 could improve endocrine and metabolic disorders (13). However, there were certain side effects, such as liposarcoma (97). On the other hand, there is limited clinical evidence, supporting the efficiency and safety of IL-22 in humans. Thus, safer therapies are needed to be developed as soon as possible.

The probiotics (or synbiotics) supplementation and fecal microbiota transplantation (FMT) might have a significant impact in this regard. Studies indicated that the administration of probiotics (or synbiotics) for 8–12 weeks could lower serum levels of glucose, insulin, triglycerides (TGs), very low–density lipoprotein, and cholesterol while improving the IR, lipid metabolic disturbance, and inflammatory state. It could also effectively lower the body weight and BMI of patients with PCOS (98–100). Nevertheless, a meta-analysis showed that the effects of probiotics (or synbiotics) supplementation on LDL, weight, BMI, and IR were not significant (101). However, probiotics had certain effects on regulating the metabolisms of glucose, insulin, and lipids, which could lower the serum levels of glucose, insulin, and TG while increasing HDL (101). Another study indicated that the supplementation of probiotics (or synbiotics) improved androgen metabolism without having any other therapeutic effect (102). The administration of different probiotic species and doses to the patients with varied PCOS phenotypes might explain the differences in these outcomes. In rodents, FMT could treat PCOS by restoring the composition of gut microbiota and improving the sex hormone balance and ovarian function (103). However, currently, there are limited applications of FMT in the treatment of PCOS. Therefore, the use of FMT in humans is yet to be determined.

The results showed that the supplementation of insulin-enriched synbiotic yogurt to the PCOS mice could decrease the body weight gain, improve estrus cycles and ovary morphology, and reduce the levels of LH while increasing those of follicle-stimulating hormone and IL-22 in serum. At the genus level, the synbiotic yogurt increased the relative abundances of *Lactobacillus*, *Bifidobacterium*, and *Akkermansia* (104).

Traditional Chinese medicines (TCMs) are vast treasure, which need to be explored. The TCMs, including flavonoids, polysaccharides, saponins, and other compounds, might possess tremendous prospects for stimulating the growth of a particular gut microbial species, increasing the production of beneficial SCFAs and BAs, and suppressing the growth of pathogenic bacteria and BCAA products (94, 105, 106). Berberine, a powerful natural product, which is used for the treatment of metabolic syndrome, could lower the abundance of BCAA-producing bacteria and aberrant blood BCAA levels in the HFD-induced rodents, thereby improving the glucose metabolism, lipid metabolism, and IR in the PCOS rodents (107, 108). Studies on the rodents demonstrated that baicalin could boost the SCFA generation and alter the BA metabolism by modifying the immunology and gut microbiota, as well as affecting the liver–gut axis by regulating the BA-FXR/TGR5 signaling pathway (109). Ginseng polysaccharides and ginsenosides could also boost the growth of *Lactobacillus* spp. and *Bacteroides* spp. in the rat. These two were the most significantly boosted probiotics, restoring the balance of gut microbiota and thereby regulating intestinal metabolism (110). Thus, the TCM and natural products might affect the gut immunity, barrier, and gut microbiota to modulate the local metabolisms. Although studies have revealed that some TCM products have low bioavailability, it is remarkable that gut microbes can transform them into components, which can be absorbed more easily, thereby improving their efficiency and indicating the positive interaction between TCM and gut microbiota (94, 111–113).

Adopting a healthier lifestyle might also improve PCOS, especially the exercise and diet, which act as the modulators of gut microbiota. As mentioned before, bad habits such as an adoration of sweets, a love of fat, an absence of dietary fiber, and little exercise, might disrupt gut health. As compared to the Western diet (enriched in animal protein and fat and low in fiber), gluten-free diet, vegetarian diets (high in fermentable plant-based foods), etc., the Mediterranean diet is more recommended for the patients with PCOS (114). Numerous human or rodent studies have demonstrated that the Western diet could significantly decrease the abundance of total and beneficial bacteria species, including *Bifidobacterium* and *Eubacterium* (115). The beneficial bacterial populations, such as *Bifidobacterium* and *Lactobacillus*, decreased, whereas those of potentially harmful bacteria increased in the human who consumed a gluten-free diet (116). The results of various studies on vegetarian diets are contradictory. In general, the Mediterranean diet is characterized as a healthy and balanced diet, which can improve obesity, lipid profile, and inflammation (114, 117). Specifically, the assorted fruits, vegetables, nuts, legumes, and cereals are recommended, whereas the intake of red meat, processed meat, and sweets should be limited (114). Exercise can enhance gut health by increasing the diversity of gut microbiota and balancing the beneficial and pathogenic bacterial communities, and a minimum of 150-min exercise of moderate intensity per week is necessary for patients with PCOS (1).

The studies on gut health and PCOS are still in the initial stages, which limit the scope of this review. On one hand, this review mainly focused on the interactions of PCOS with the SCFAs, BAs, and amino acids. Nevertheless, there might be additional metabolic loops closely associated with the PCOS, such as carnitine metabolism (118). The amino acids, other than BCAAs, are also needed to be thoroughly investigated in the future. On the other hand, there might be some changes in the synthesis and transit of gastrointestinal metabolites between species. Therefore, the findings from animal studies remain to be validated in humans.

## Author Contributions

MZ and YS contributed to the conception of this review. RH wrote the manuscript. RH, MZ, and YS revised the manuscript. RH and YH designed and illustrated the figures. YH, FZ, FL, ZL, and YG performed the literature search and interpretation. MZ, RH, YH, FZ, FL, ZL, YG, HD, WM, KS, and YS reviewed the manuscript. All authors approved the submission.

## Conflict of Interest

The authors declare that the research was conducted in the absence of any commercial or financial relationships that could be construed as a potential conflict of interest.

## Publisher’s Note

All claims expressed in this article are solely those of the authors and do not necessarily represent those of their affiliated organizations, or those of the publisher, the editors and the reviewers. Any product that may be evaluated in this article, or claim that may be made by its manufacturer, is not guaranteed or endorsed by the publisher.
